# Supplementary material for: Fabrication and Characterization of Dielectric ZnCr2O4 Nanopowders and Thin Films for Parallel-Plate Capacitor Applications
Source: Micromachines (Basel). 2023 Sep 12;14(9):1759. doi: 10.3390/mi14091759 (PMC10534308; doi:10.3390/mi14091759)
Supplement: Supplementary file 1 [file micromachines-14-01759-s001.zip › micromachines-2513474-supplementary.pdf]

Supplementary information

Fabrication and Characterization of Dielectric $ZnCr_2O_4$ Nanopowders and Thin Films for Planar Capacitor Applications

Vasyl Mykhailovych[1-5], Gabriel Caruntu[1-3,], Adrian Graur[1], Mariia Mykhailovych[1,4], Petro Fochuk[4], Igor Fodchuk[5], Gelu-Marius Rotaru[6], Aurelian Rotaru[1,*]*

[1]Department of Electrical Engineering and Computer Science & Research Center MANSiD, Stefan cel Mare University of Suceava, Strada Universității 13, 720229, Suceava, Romania

[2]Department of Chemistry and Biochemistry, Central Michigan University, 1200 S. Franklin St., Mount Pleasant, MI 48859, USA

[3]Science of Advanced Materials Program, Central Michigan University, 1200 S. Franklin St., Mount Pleasant, MI 48859, USA

[4]Department of General Chemistry and Material Science, Yuriy Fedkovych Chernivtsi National University, 2, Kotsjubynskyi St., 58012, Chernivtsi, Ukraine

[5]Physical, Technical and Computer Science Institute, Yuriy Fedkovych Chernivtsi National University, 2, Kotsjubynskyi St., 58012, Chernivtsi, Ukraine

[6]Faculty of Mechanical Engineering Mechatronics and Management & Research Center MANSiD, Stefan cel Mare University, 720229 Suceava, Romania

* Correspondence: g.caruntu@cmich.edu and aurelian.rotaru@usm.ro

Keywords: High-k material, $ZnCr_2O_4$ nanoparticles; shape-controlled synthesis; dielectric properties; thin films; planar capacitor.

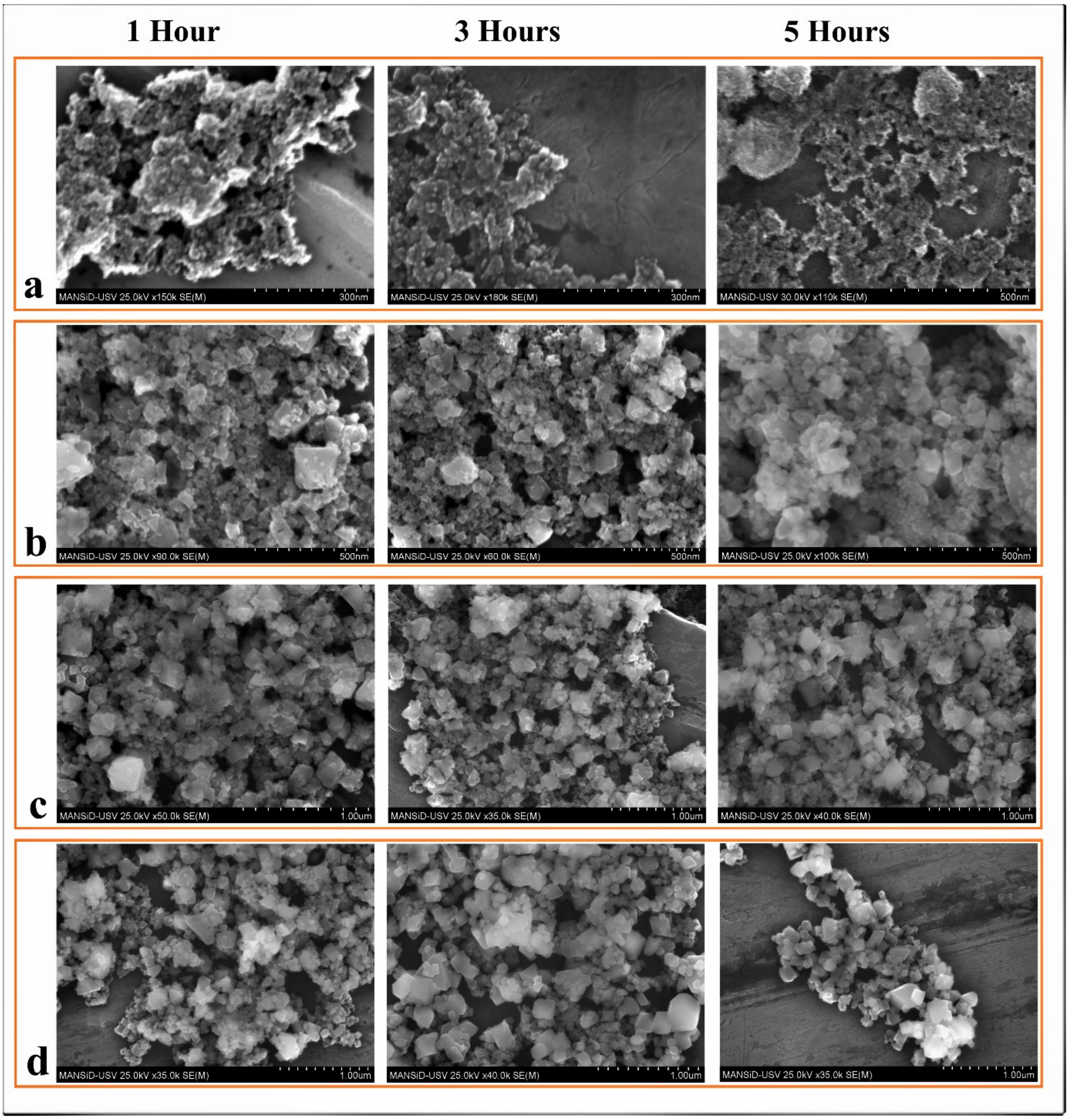

Figure S1. Electron microscopy images recorded on $ZnCr_2O_4$ nanoparticles annealed at 500 ºC, 700 ºC, 800 ºC and 900 ºC for 1, 3 and 5 hours respectively.

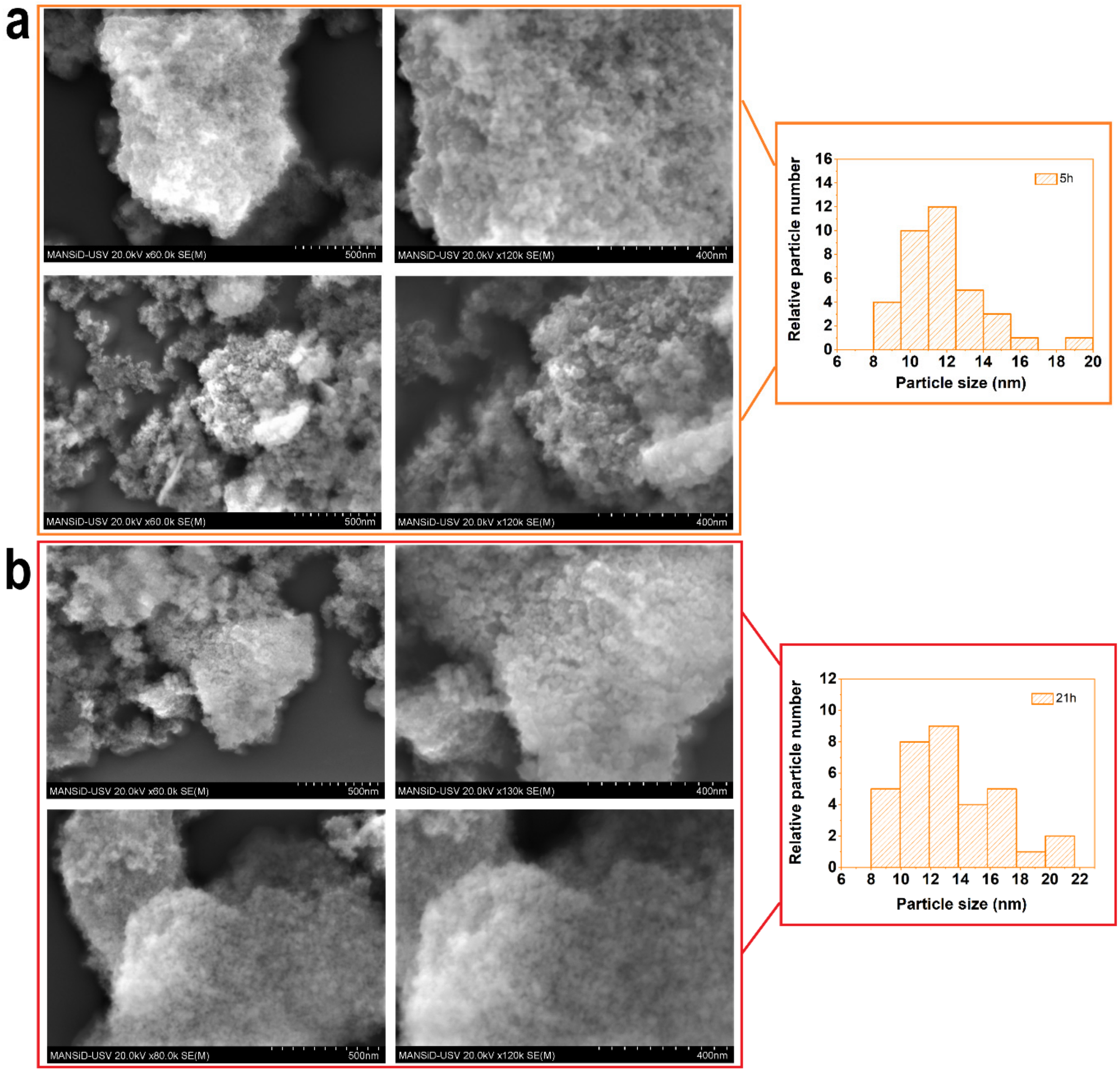

Figure S2. Series of electron microscopy images recorded on $ZnCr_2O_4$ nanoparticles annealed at 500 °C for: **(a)** 5 hours and **(b)** 21 hours with their corresponding size distribution histograms.

.

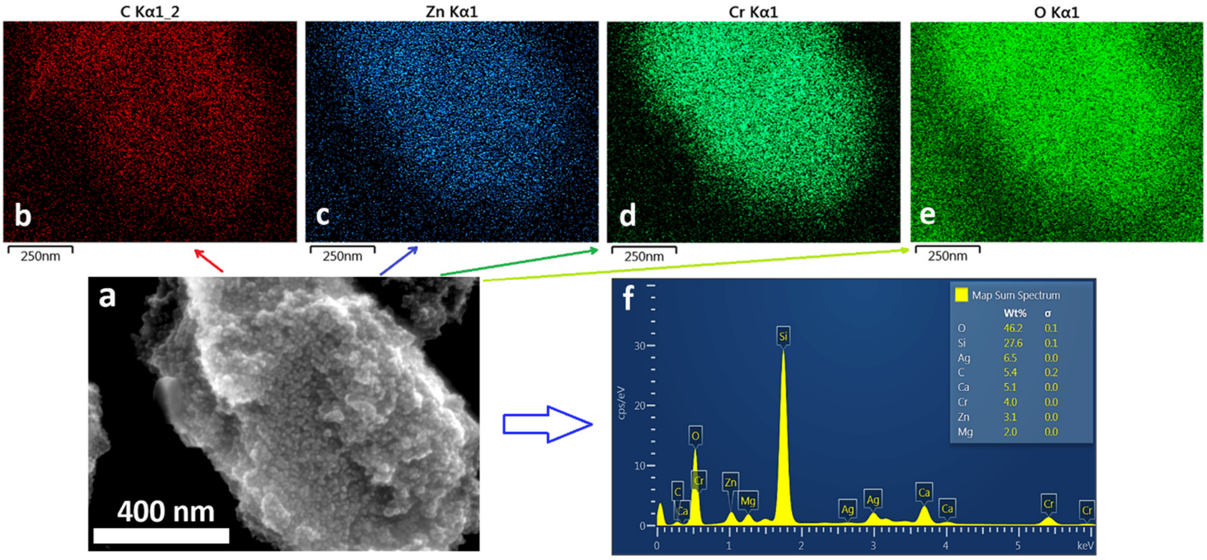

Figure S3. Depict FE-SEM image of sample annealed for 5 hours at 500 °C – (a), elemental mapping (b-e) and EDX spectrum with weight distribution of elements – (f).

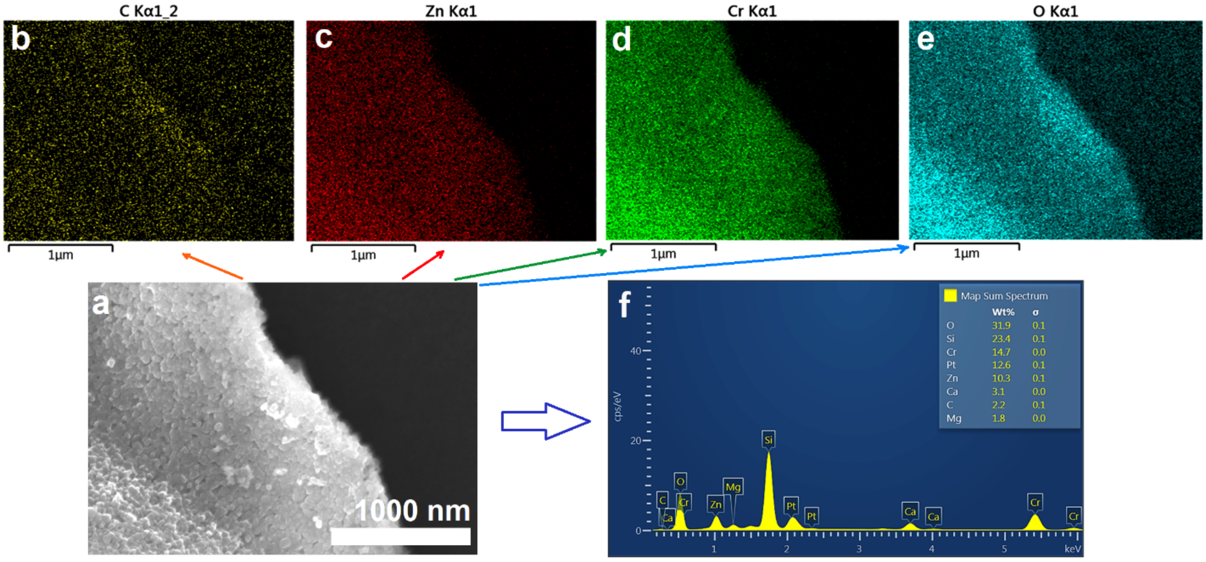

Figure S4. Depict FE-SEM image of sample annealed for 7 hours at 700 °C – (a), elemental mapping (b-d) and EDX spectrum with weight distribution of elements – (e).

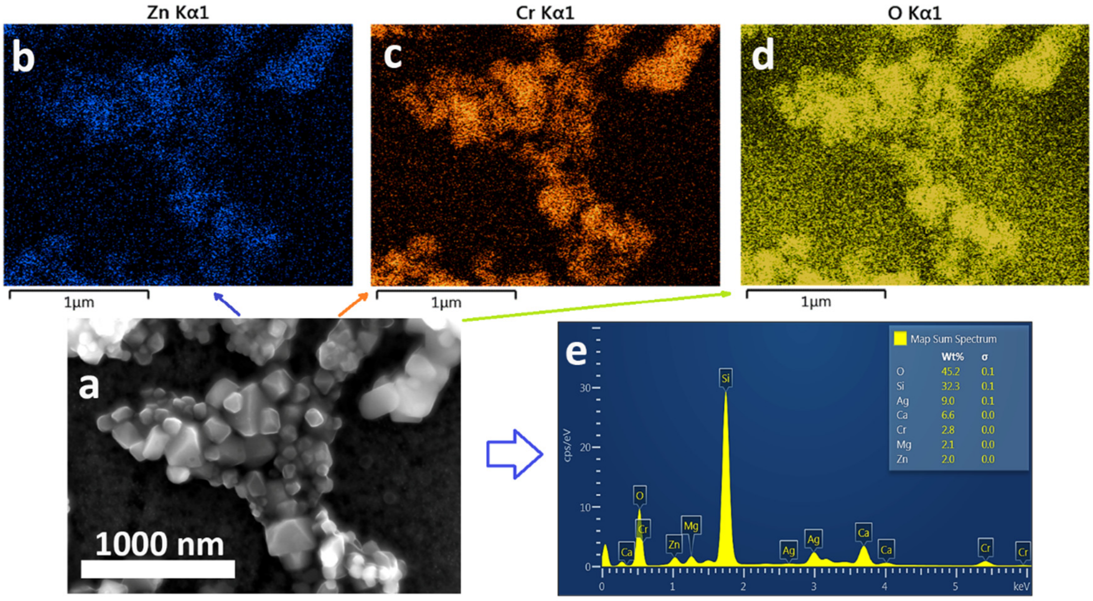

Figure S5. Depict FE-SEM image of sample annealed for 9 hours at 800 °C – (a), elemental mapping (b-d) and EDX spectrum with weight distribution of elements(e).

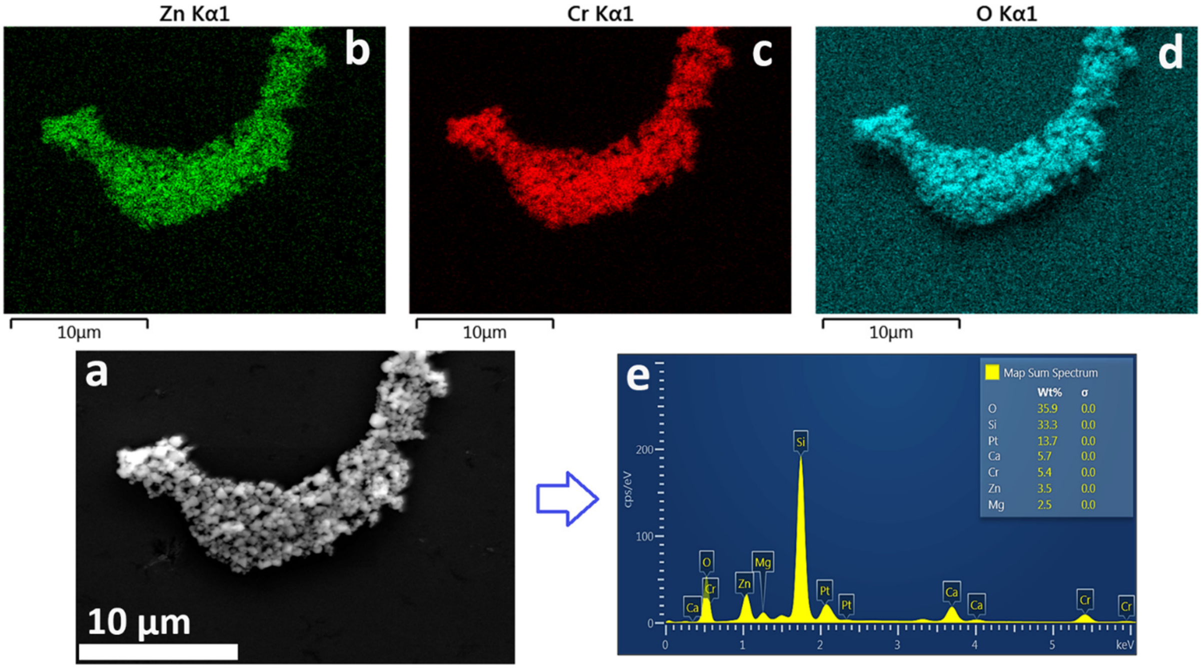

Figure S6. Depict FE-SEM image of sample annealed for 11 hours at 900 °C – (a), elemental mapping (b-d) and EDX spectrum with weight distribution of elements – (e).

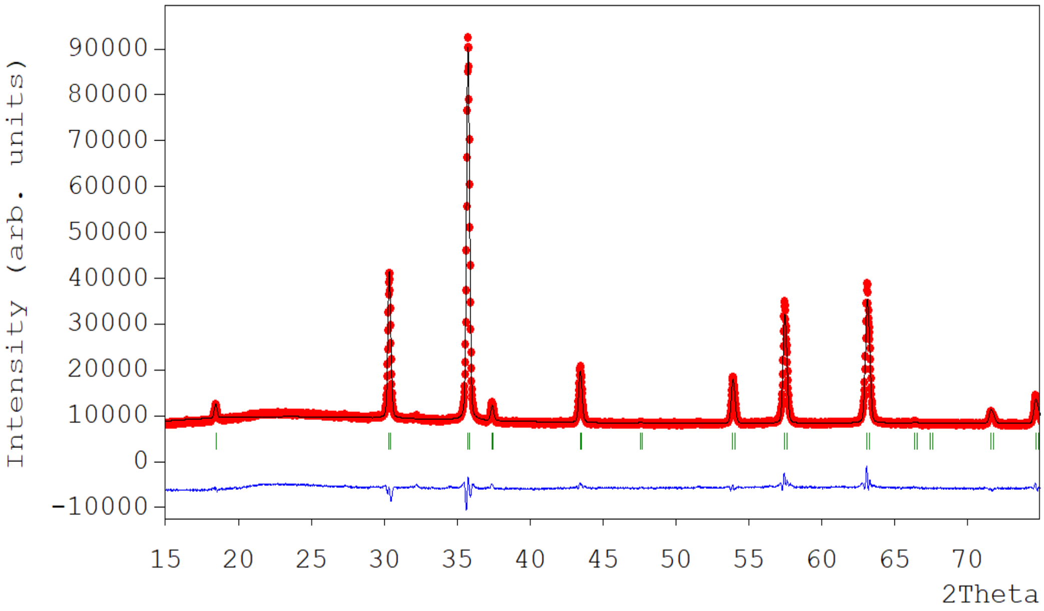

Figure S7. Rietveld refinement plots of nanostructured $ZnCr_2O_4$ samples annealed in air at 800°C for 9h. The blue curve represents the difference I_{obs}-I_{calcd} pattern, whereas the green vertical bars represent the positions of the Bragg reflections

Table S1. Crystallographic parameters obtained from the Rietveld refinement of the ZnCr2O4 nanopowder annealed at 800 oC in air for 9 hours

Phase	Lattice Parameters (Å)	Atomic Coordinates			Occupancy	B_{iso}	R factors
ZnCr2O4	a=b=c	Ion	x=y=z	Wyckoff			R_p=2.81%; R_{wp}=3.75%, R_{exp}=1.90, χ^2=13.6
	8.328(4)	Zn2+	0.125	8a	1.0	0.72	
		Cr3+	0.500	16d	1.0	0.45	
		O2-	0.259(1)	32e	1.0	0.79	

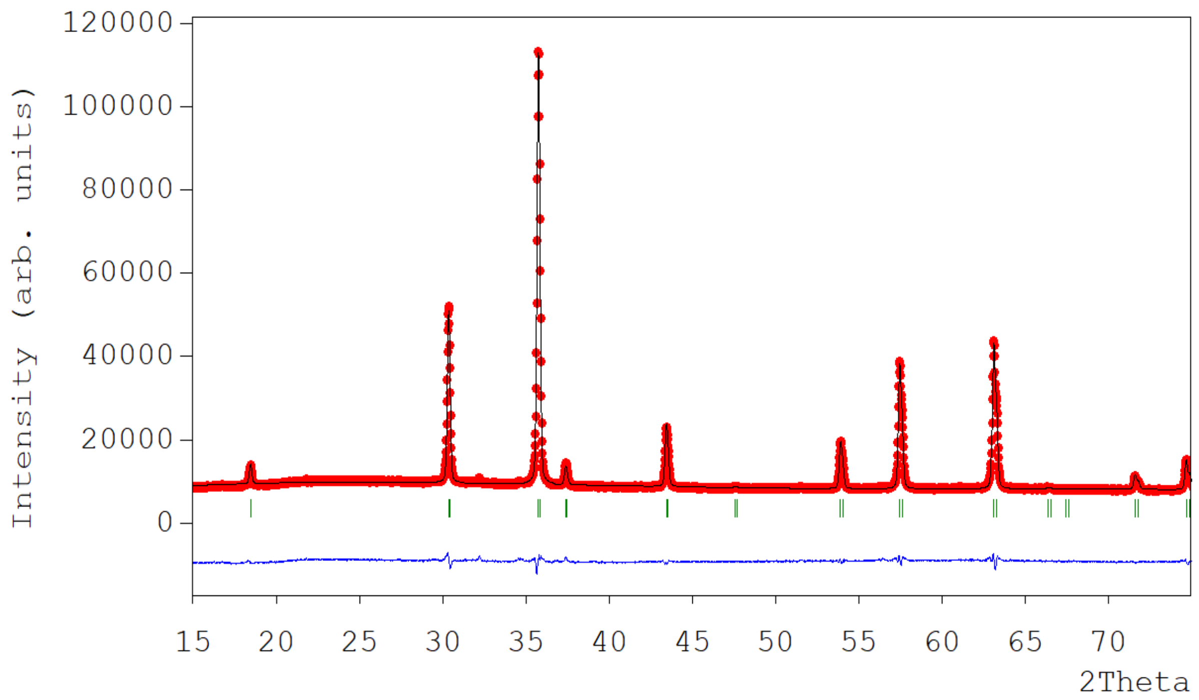

Figure S8. Rietveld refinement plots of nanostructured $ZnCr_2O_4$ samples annealed in air at 900°C for 11h. The blue curve represents the difference I_{obs}-I_{calcd} pattern, whereas the green vertical bars represent the positions of the Bragg reflections

Table S2. Crystallographic parameters obtained from the Rietveld refinement of the ZnCr2O4 nanopowder annealed at 900 °C in air for 11 hours

Phase	Lattice Parameters (Å)	Atomic Coordinates			Occupancy	B_{iso}	R factors
ZnCr2O4	a=b=c	Ion	x=y=z	Wyckoff			R_p=2.37%; R_{wp}=2.95%, R_{exp}=1.01, χ^2=8.52
	8.328(4)	Zn2+	0.125	8a	1.0	0.72	
		Cr3+	0.500	16d	1.0	0.45	
		O2-	0.259(8)	32e	1.0	0.79	
